# Supplementary material for: Nutritional Risk and Sarcopenia Features in Patients with Crohn’s Disease: Relation to Body Composition, Physical Performance, Nutritional Questionnaires and Biomarkers
Source: Nutrients. 2023 Aug 17;15(16):3615. doi: 10.3390/nu15163615 (PMC10458234; doi:10.3390/nu15163615)
Supplement: Supplementary file 1 [file nutrients-15-03615-s001.zip › nutrients-2507207-supplementary.pdf]

Supplementary Table S1: Spearman correlation coefficients between MNA score, MNA sub-scores and body composition/function patterns in patients with remission (subsample of the present study).

	Pattern 1 (MAC, FFMI, Max HGS)	Pattern 2 (Gait speed)	Pattern 3 (CC, MAC, age)	Pattern 4 (Chair-standing test, OLST)	Pattern 5 (Alb, TSF)
MNA Total score	0.215	0.110	**0.417**	0.066	0.178
	p=0.312	p=0.608	**p=0.043**	p=0.761	p=0.405
MNA Food intake	0.306	0.092	0.332	0.366	**0.455**
	p=0.147	p=0.670	p=0.113	p=0.078	**p=0.025**
MNA weight loss	**-0.404**	0.291	-0.048	-0.162	0.210
	**p=0.050**	p=0.168	p=0.822	p=0.451	p=0.325
MNA mobility	NA	NA	NA	NA	NA
MNA acute stress	0.044	0.106	0.367	0.180	0.193
	p=0.840	p=0.623	p=0.078	p=0.399	p=0.367
MNA neurological problems	0.256	-0.045	0.346	-0.166	-0.166
	p=0.227	p=0.834	p=0.097	p=0.439	p=0.439
MNA BMI	**0.700**	-0.112	0.347	-0.045	0.116
	**p<0.001**	p=0.602	p=0.097	p=0.836	p=0.588

MAC: Mid arm circumference; FFMI: Fat free mass index; HGS: hand-grip strength; CC: Calf circumference; OLST: One-Legged Stance Test; Alb: Albumin; TSF: Triceps Skinfold; MNA: Mini Nutritional Assessment; BMI: Body Mass Index

NA: Not applicable. It is noted that no variability was detected for MNA-mobility and thus no correlation coefficient could be calculated.
